# Supplementary material for: Disentangling metabolic functions of bacteria in the honey bee gut
Source: PLoS Biol. 2017 Dec 12;15(12):e2003467. doi: 10.1371/journal.pbio.2003467 (PMC5726620; doi:10.1371/journal.pbio.2003467)
Supplement: S8 Data — (ZIP) [file pbio.2003467.s008.zip › Z-score_plots/568 microbial substrate (9Z)-(13S)-12,13-Epoxyoctadeca-9,11-dienoic acid.pdf]

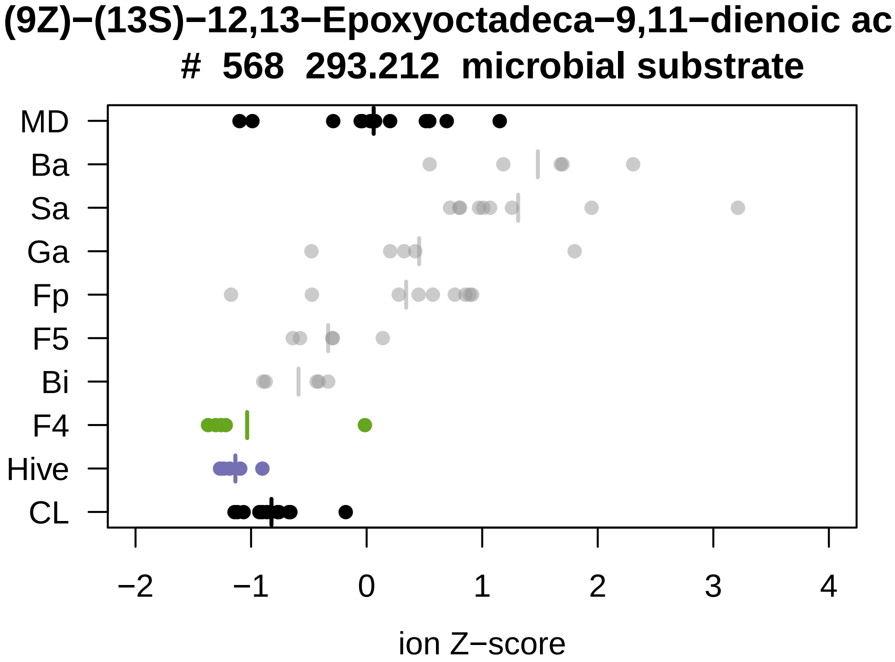

(9Z)−(13S)−12,13−Epoxyoctadeca−9,11−dienoic ac
568 293.212 microbial substrate
MD
Ba
Sa
Ga
Fp
F5
Bi
F4
Hive
CL
−2
−1
0
1
2
3
4
ion Z−score
